# Combined gas well hydrate prevention and control technology and its application

**DOI:** 10.1371/journal.pone.0295356

**Published:** 2023-12-07

**Authors:** Leilei Gong, Shujin Zhang, Meng Cai, Junliang Li, Qiuyu Lu, Xiaochuan Zhang

**Affiliations:** 1 Northeast Petroleum University, Daqing, China; 2 Research Institute of Oil Production Engineering, Daqing Oilfield Co., Daqing, China; 3 Heilongjiang Oil and gas Reservoir Production and Increase Focus Laboratory, Daqing, Heilongjiang Province, China; China University of Mining and Technology, CHINA

## Abstract

The high pressure in some gas wells, such as those in the Xushen gas field in Daqing, China, makes them susceptible to freezing and hydrate blockages. Downhole throttling technology is widely used to reduce costs during well construction, however, due to the limitations of temperature, pressure and depth structure, this technology is sometime applied independently in some gas wells in which freezing and blockages are a frequent problem that can seriously affect production capacity. Moreover, artificial alcohol injection of ‘passive plugging’ to prevent hydrate formation not only consumes significant amounts of methanol but its efficiency is also dependent on factors such as weather, personnel and equipment, so it is not a continuous solution. In order to solve the above problems, the mechanism of hydrate formation was analyzed in this study, from which a combined mechanical and chemical hydrate control process was developed. OLGA software was used to design the process parameters of the novel mechanical and chemical inhibition technology for hydrate prevention and control, and also to simulate and analyze the wellhead temperature, pressure and hydrate generation once the process was implemented. Based on the results of the parameters calculation, the downhole throttle and hydrate inhibitor automatic filling device are used to realize the functions of downhole throttle depressurization and hydrate inhibitor continuous filling, reduce the wellhead pressure and hydrate generation temperature, and ensure the continuous production of gas well. This novel combination process was subsequently tested in three wells in the Daqing gas oilfield. Measurements showed that the average daily gas increase from a single well was 0.5×10^4^m^3^, methanol consumption was reduced from the original maximum daily amount of 1750 kg to just 60 kg, the manual maintenance workload was reduced by 80%, and the rate of the well openings was increased from 45% to 100%. These results proved that this technology is feasible and efficient for applications in gas wells with high downhole pressure and low wellhead temperature, and, thus, provides important technical support for the prevention of gas hydrate and improvement of gas well production.

## 1 Introduction

With the increasing demand for oil and natural gas, the oil and gas industry has been developing rapidly, and the exploration target layer has extended from the middle and shallow layer to the deep and ultra-deep layer, and the deep layer will become one of the important development fields of the oil field industry [1]. As an important special oil and gas reservoir, volcanic rocks have been found in more than 300 basins and regions in more than 20 countries. In recent years, with the breakthrough in the exploration of volcanic gas reservoirs in Songliao Basin, Xujiaweizi fault depression has become the fault depression with the highest degree of deep exploration and the most gas reserves found in the northern Songliao Basin, and it is the fifth largest gas bearing area on land in China. Xushen Gas field has become the volcanic gas field with the largest proven reserves of natural gas in China.However, there is an important problem in the development process of natural gas. As natural gas is a compressible fluid, the pressure of wellhead and transmission line will be too high in the mining process, resulting in many safety risks. In order to solve this problem, downhole throttling technology can be adopted to achieve wellhead pressure reduction, solve hydrate freezing blockage, improve stable production capacity and reduce well construction cost.

In a gas well, downhole throttling technology involves the placement of special equipment at an appropriate position in the production tubing to realize wellbore throttling and depressurization [2], thereby enabling the surface production pipeline to operate at a lower pressure. Heat formed in the gas well is utilized to compensate for the temperature drop generated by the wellbore after throttling, effectively preventing the formation of hydrate in the wellbore and surface pipeline during production. Many scholars have carried out research on downhole throttles, Hu Dan et al. [3] developed movable downhole throttles characterized by a short length, small exterior diameter, smooth delivery, reliable release, a latch and seal feature, and a high salvage success rate, which aimed to alleviate fishing difficulties and to improve the low success rate of early downhole throttles used in the western Sichuan gas field. Wang Yifei et al. [4] established a new model in which the gas-liquid mixture flowed through the nozzle, and compiled calculation software for the downhole throttling process parameters of water-bearing gas wells that improved the reliability of the process. Wu Yibo et al. [5] proposed an improved ‘V’-type cylindrical rubber structure for which a finite element analysis model was also established. Improves the sealing of the rubber cylinder. Zhou Jian [6] introduced “slippage factor K” to characterize the slippage effect between gas and liquid, optimized the pressure drop model of the gas-liquid two-phase nozzle flow, and established an method for optimizing the downhole throttling process parameters of liquid-producing horizontal wells. Yuan Hang et al. [7] analyzed the application conditions for downhole throttling technology in high-pressure shale gas wells in the south Pingqiao area of China, while also comparing variations in the gas volume and pressure in a single well before and after the application of the technology, and determining the running depth, aperture and timing of downhole throttling.

Compared with the traditional surface throttling process, downhole throttling provides several advantages, including a reduction in the operating pressure of the surface pipeline, reduction of the investment cost of ground equipment, and an improvement in the rate at which the well opens. However, for some special types of high-pressure gas Wells, due to the limitations of temperature, pressure and well structure of some gas Wells, it is impossible to realize the throttling pressure reduction and inhibit hydrate formation under critical flow conditions.

At present, chemical control technology is commonly used for hydrate control, both in China and abroad. Chemical hydrate prevention technology includes the application of an automatic drip device to automatically and continuously inject hydrate inhibitor into the gas well, according to specific injection intervals and amounts as calculated via the design. Hydrate inhibitor is a chemical substance that inhibits the formation of hydrate during the exploitation, transportation and treatment of natural gas. The addition of such inhibitors to gas wells can change the chemical potential of an aqueous solution or hydrate phase, so that hydrate formation conditions are shifted to a lower temperature or higher pressure range. Domestic and foreign scholars have conducted relevant studies on the action principle of hydrate inhibitors. Ng et al. [8] found that the addition of methanol and electrolyte can effectively inhibit the formation and accelerate the decomposition of hydrate through the study of hydrate. Kotkoski et al. [9] found that injection of methanol or glycerol could inhibit hydrate formation. After the experiment of injecting glycol into CH_4_ hydrate, Fan et al. [10] concluded that the dissociation rate of CH_4_ hydrate depends on the concentration and flow rate of glycol. Dong et al. [11] studied C_3_H_8_ hydrate injected with methanol and found that methanol could reduce the dissociation heat of C_3_H_8_ hydrate and increase its dissociation rate compared with pure water injection. Wan Lihua et al. [12] revealed the effect of ethylene glycol on the decomposition of CH_4_ hydrate. Yagasaki et al. [13] studied the mechanism of methanol inhibition by MD simulation method, and the results showed that the hydrate decomposition rate was not a constant value. The addition of methanol increased the decomposition rate of CH_4_ hydrate, and the role of methanol molecules was to help generate bubbles to carry away CH_4_ molecules. However, the chemical control method of hydrate alone can not reduce the wellbore pressure, so it is necessary to use high pressure wellhead and high pressure pipeline, which has high cost of well construction and low economic benefit of high pressure and low gas production Wells.

In view of the limitations of the two existing technologies for hydrate control in deep high pressure and low gas production Wells, this paper proposes a hydrate combination control technology, which combines downhole throttling technology and hydrate chemical control technology. Downhole throttling technology can reduce throttling pressure, hydrate chemical control can inhibit hydrate formation, reduce oil pressure and prevent hydrate freezing blockage in special high-pressure gas Wells at the same time, and improve the opening rate and stable production capacity of such gas Wells.

## 2 Mechanism of hydrate formation

**Fig 1:**
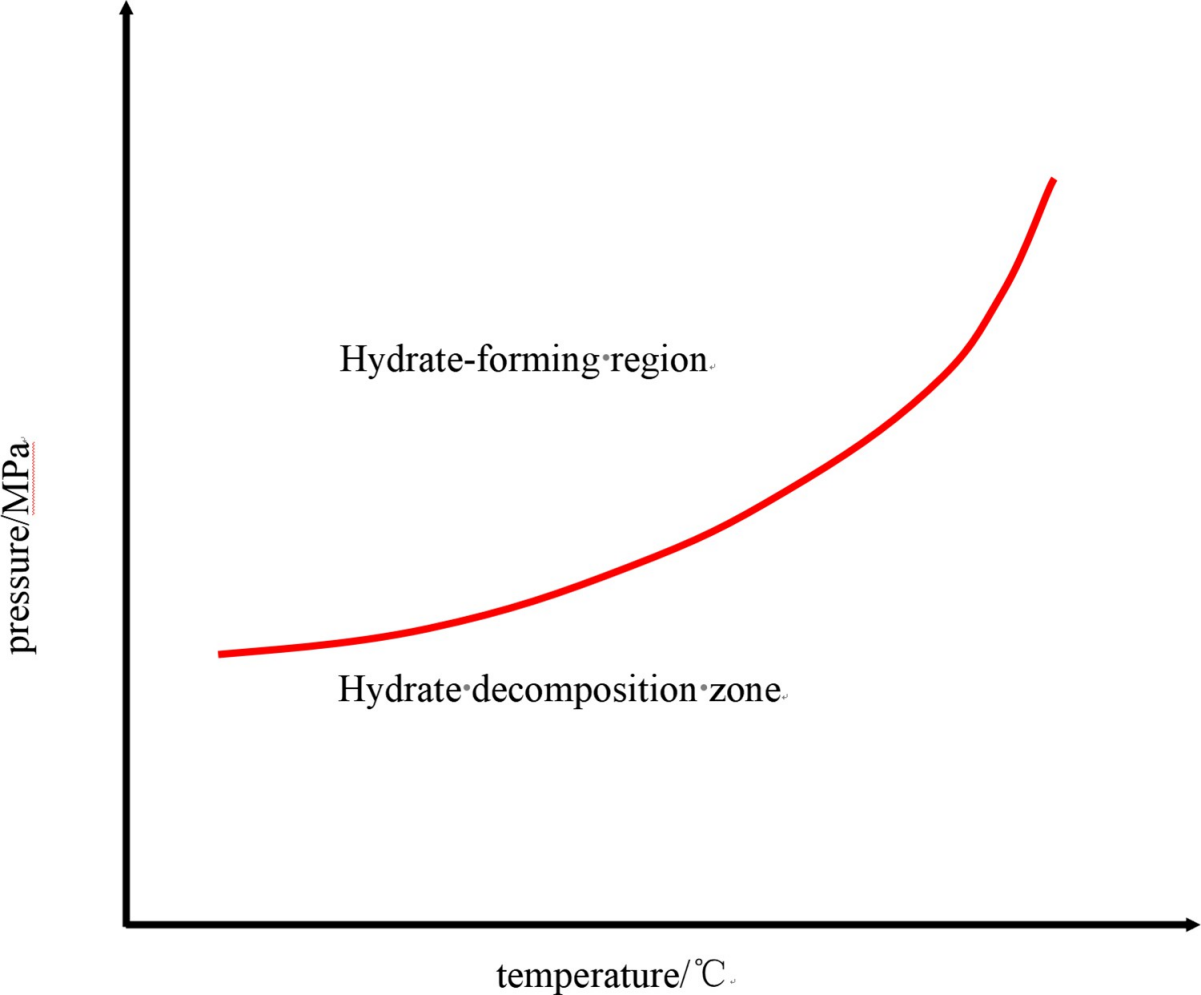
Phase equilibrium curve of hydrate.

Hydrate generation in gas wells is mainly affected by the temperature and pressure distribution of the fluid and fluid composition, among other factors [14]. Hasan et al. [15] and Wang et al. [16] proposed models through which to calculate changes in the temperature and pressure fields in the wellbores of gas wells, while the Chen-Guo model [17] is widely used to calculate the temperature and pressure during gas hydrate phase equilibrium under multi-component conditions. A curve diagram can be generated to show the effect of temperature and pressure on hydrate generation at each position in the wellbore, as shown in Fig 1, in which it is evident that the conditions for hydrate generation are satisfied either when the fluid temperature is lower than the temperature of the phase equilibrium point or when the pressure is higher than the phase equilibrium point. The intersecting area between the flow temperature pressure curve and the hydrate phase equilibrium curve in Fig 1 is the hydrate generation area, and the transverse width of this area represents the degree of undercooling for hydrate generation. The greater the degree of undercooling, the greater the probability and, thus, risk of hydrate generation. Since the formation of hydrate is influenced by the temperature and pressure inside the wellbore, these two factors in the wellbore environment must be managed to inhibit the formation of hydrate.

While the temperature of fluid in a gas well gradually decreases, water vapor in various positions in the wellbore changes simultaneously from a saturated to an unsaturated state, continuously condensing and resulting in the precipitation of free water on the wall of the radial cold pipe and, thus, a source for the formation and deposition of hydrate. The transient wellbore temperature distribution in the phase of decreasing fluid temperature can be calculated as follows [18]:

$$T_{f}=\left(T_{f0}-T_{ei}\right)•e^{-\frac{L^{'}R}{m{\left(1+C_{T}\right)}^{t}}}+T_{ei}$$ (1)

Where T_f_ is wellbore fluid temperature, K; T_f0_ is wellbore fluid temperature at initial shut-in time, K; T_ei_ is ambient temperature, K; t is off-time, s; L’_R_ is shut-in relaxation parameters, kg/(m·s); m is mass of fluid per unit length, kg/m; C_T_ is coefficient of wellbore heat storage.

When the temperature and pressure conditions of hydrate formation are satisfied in the wellbore, the free water precipitated from condensation on the pipe wall is converted into hydrate. Since the tube wall exerts a strong adhesive force on the hydrate generated upon its surface, with no external force influencing the adhesion process, all the hydrate generated by condensate water will remain deposited on the tube wall, so that the hydrate deposition rate is equal to rate of its formation. The rate of free water precipitation generated from oversaturation while both fluid temperature and radial temperature are gradually decreasing can be obtained by calculating the change in natural gas water saturation at different times and positions during the shut-in period. According to the law of conservation of mass, the hydrate formation and deposition rates in the radial temperature drop stage can be obtained as follows [19]:

$$R_{dh}=\frac{M_{h}}{5.75M_{w}}•\frac{d(C_{ws}V_{e}\frac{ZT_{0}p}{Tp_{0}})}{\mathrm{dt}}$$ (2)

$$C_{\mathrm{ws}}=101.325\sum_{k=0}^{7}\frac{a_{k}{\left(T-273.15\right)}^{k}}{p}+\sum_{k=0}^{7}b_{k}{\left(T-273.15\right)}^{k}$$ (3)

Where R_dh_ is hydrate deposition rate, kg/s; M_h_ is molar mass of hydrate, kg/mol;M_w_ is molar mass of water, kg/mol; C_ws_ is natural gas water saturation, kg/m^3^; V_e_ is active volume, m^3^; Z is natural gas compression factor; T_0_ is reduced temperature, 288.15 K; p is system pressure, MPa; T is natural gas water dew point temperature, K; p_0_ is barometric pressure, 0.101MPa; a,b is solution coefficient of natural gas water saturation.

When the fluid temperature in the wellbore reaches that of the formation temperature outside, the concentration of saturated water molecules at various positions will be different due to the difference in axial temperature and pressure in the wellbore. Under the action of molecular diffusion force, water molecules will diffuse from the high concentration area to the low concentration area in a mass transfer process [20], causing water molecules in the low concentration zone to change from a saturated to an unsaturated state and then condensing into free water. This provides a free water source for the formation of hydrate, which is deposited on the tube wall and forms a hydrate layer. According to the law of mass conservation, the hydrate formation and deposition rate in this case can be obtained as follows [21]:

$$R_{dh}=\frac{M_{h}}{5.75M_{w}}•(-D_{AB}\frac{\partial C_{w}}{\partial x})•A_{e}$$ (4)

$$D_{AB}=\frac{1.00\times 10^{-3}T^{1.75}{\left(1/M_{A}+1/M_{B}\right)}^{1/2}}{p_{a}•{\left[{\left(\sum {}_{A}V_{i}\right)}^{1/3}+{\left(\sum {}_{B}V_{i}\right)}^{1/3}\right]}^{2}}$$ (5)

Where D_AB_ is diffusion coefficient between water molecules and methane, m^2^/s; $\frac{\partial C_{w}}{\partial x}$ is axial water molecular concentration gradient, kg/m^4^; A_e_ is effective flow area, m^2^; M_A,_M_B_ is molar mass, kg/mol; p_a_ is pressure, MPa; $\sum_{A}V_{i}、\sum_{B}V_{i}$ is diffusion volume of components A and B, cm^3^/mol.

By contrast, when the temperature of fluid at different positions in the wellbore of a gas well increases until the stability of the hydrate can no longer be maintained, any previously formed hydrate sedimentary layer deposited on the wall of the pipe will gradually decompose into gas and water, thereby reducing the hydrate in the wellbore. Due to the differences in temperatures as well as in the thickness of the hydrate deposition layers around the wellbore, the rate of hydrate decomposition changes dynamically depending on time and location. The hydrate decomposition rate can be calculated via the following formula proposed by Goel et al. [22]:

$$-R_{\mathrm{dh}}=\pi M_{h}K_{d}{\left(f_{e}-f_{g}\right)}^{n}{\left(r_{ti}-\delta \right)}^{2}$$ (6)

Where K_d_ is hydrate decomposition constant, mol/(m^2^·Pa·s); f_e_ is three-phase equilibrium fugacity, Pa; f_g_ is gas fugacity, Pa; δ is thickness of hydrate deposit, m; r_ti_ is initial string bore, m.

The critical pressure of gas hydrate formation can be calculated according to the fluid temperature under different working conditions. When T≤273.15K, the critical pressure of hydrate formation decreases with the increase of critical temperature. When T > 273.15K, the critical pressure of hydrate formation increases with the increase of critical temperature [23].

When T>273.15K

$$\mathrm{lg}p=-1.0055+0.0541(B+\frac{T}{K}-273.15)$$ (7)

When T≤273.15K

$$\mathrm{lg}p=-1.0055+0.0171(B_{1}-\frac{T}{K}+273.15)$$ (8)

Where p is Natural gas pressure, MPa;T is the temperature of natural gas, K; B and B_1_ are dimensionless constants.

## 3. Combination hydrate control technology research and application

### 3.1 Application limits and modeling methods of hydrate combination control process

The main parameters of the combined hydrate control process include the depth of throttle, the diameter of the throttle nozzle and the amount of hydrate inhibitor injected. First, the minimum running depth of the throttle must be calculated using the formula for the minimum running depth (Formula 9, below). This measurement is then combined with those relevant to the well structure and the actual depth of the well, from which a preliminary judgment is made as to whether the running depth of the downhole throttle is sufficient the minimum running depth of the throttle. If so, the downhole throttling process can be adopted, but if not then the combined hydrate control technology would be the more suitable approach. On this basis, OLGA multiphase flow simulator software was used in this study to calculate different throttle diameters. Based on both critical flow theory and hydrate growth trend, the size of throttle diameters and the amount of hydrate inhibitors were finally determined.

Formula for minimum running depth of downhole choke:

$$L_{\min}\geq M_{0}{[(t}_{h}{+273)\beta }^{‐Z(K‐1)/K}{‐(t}_{0}+273)]$$ (9)

Where L_min_ is minimum running depth of choke, m; M_0_ is earth temperature increase rate, m/°C; t_h_ is hydrate formation temperature,°C; t_0_ is mean surface temperature,°C; β_k_ is critical pressure ratio; k is adiabatic coefficient of natural gas.

**Fig 2:**
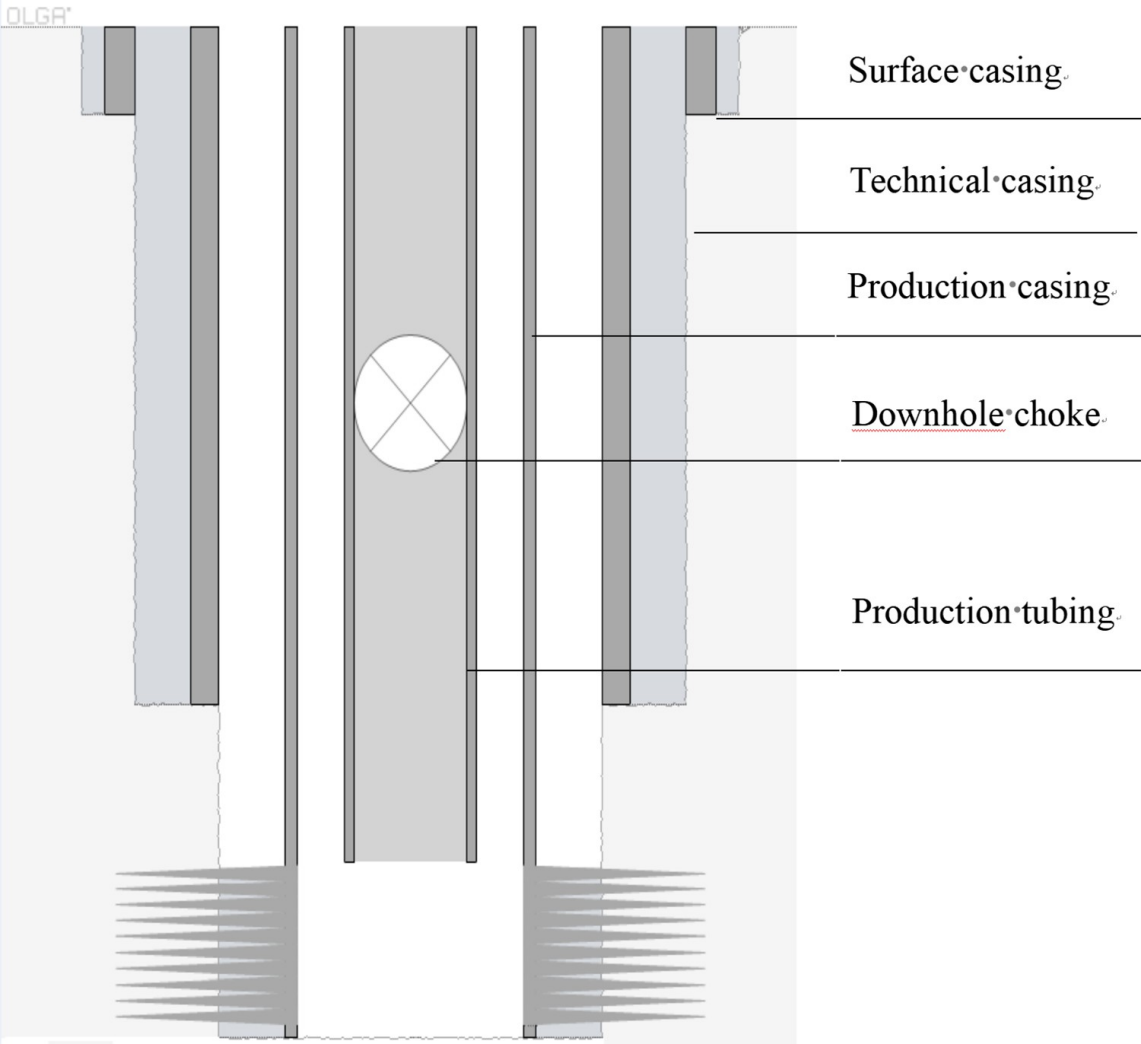
Schematic diagram of well structure.

**Fig 3:**
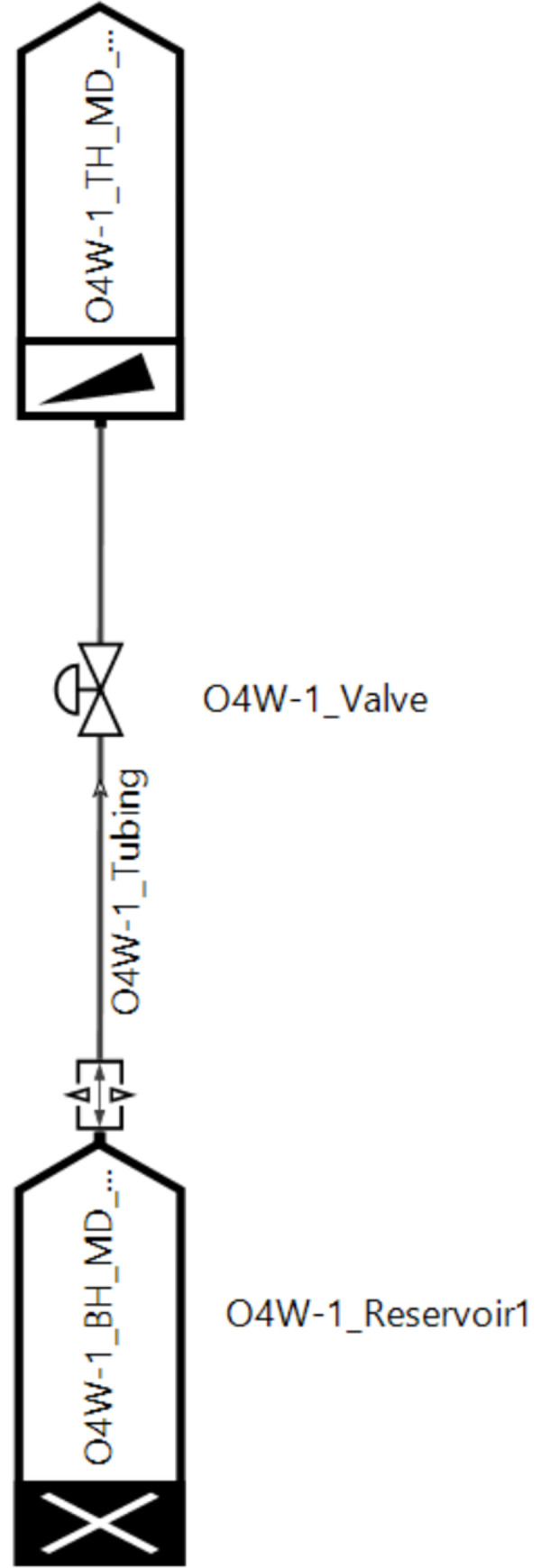
Schematic diagram of calculation model.

OLGA software can simulate the production dynamics of the gas well, obtain the temperature and pressure profile of the entire gas well wellbore before and after throttling and the trend of hydrate growth, and calculate the process parameters of hydrate combination prevention and control, which can effectively guide the on-site construction of the process. The modeling process is as follows:

Step 1: Input well structure parameters, including surface casing, technical casing, production casing, tubing and downhole choke size specifications, running depth;

Step 2: Establish the well model according to the input well structure parameters, as shown in Fig 2;

Step 3: Input formation parameters, including bottom hole temperature and pressure, reservoir parameters, wellhead temperature and pressure, wellbore static temperature and static pressure, etc., to form a calculation model. As shown in Fig 3;

Step 4: Perform dynamic simulation calculation of gas well production, simulate the real gas well production coefficient, and then obtain the temperature and pressure profile of the gas well shaft and the trend of hydrate growth.

### 3.2 Determination of process parameters of hydrate combination control

Taking well H as an example, the minimum running depth Formula (9) is applied to calculate that the minimum running depth of the choke in this well is 1496m. Since there is a landing nipple in well H at 1300m, it is determined that the hydrate combination control technology should be adopted. In the OLGA software, after simulating the establishment of the gas well production system, the running depth was set to 1300m, and according to the gas well production allocation and a large number of numerical calculations, the calculation examples of throttle diameter 1 to 5 were set to 2.4mm, 3mm, 3.6mm, 4.2mm and 4.8mm respectively, as shown in Table 1.

**Table 1:** Examples of downhole throttling parameters.

Number of throttling parameter example	1.ppl	2.ppl	3.ppl	4.ppl	5.ppl
Depth (m)	1300	1300	1300	1300	1300
Mouth diameter (mm)	2.4	3	3.6	4.2	4.8

**Fig 4:**
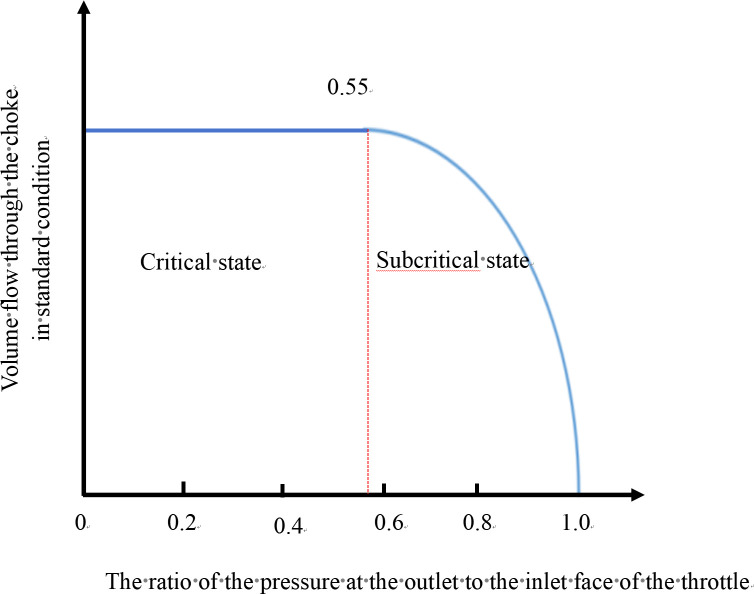
Relational diagram of volume flow rate and pressure ratio of throttle nozzle.

When the ratio of pressure between the outlet orifice and the inlet face of the throttling device is less than 0.55, the throttle is in a critical flow state and the fluctuation of pressure from behind does not affect the front of the throttle. At this time, the gas flow through the throttle is at its maximum [24], as shown in Fig 4. Therefore, it is necessary to achieve throttling pressure reduction under critical flow conditions.

**Fig 5:**
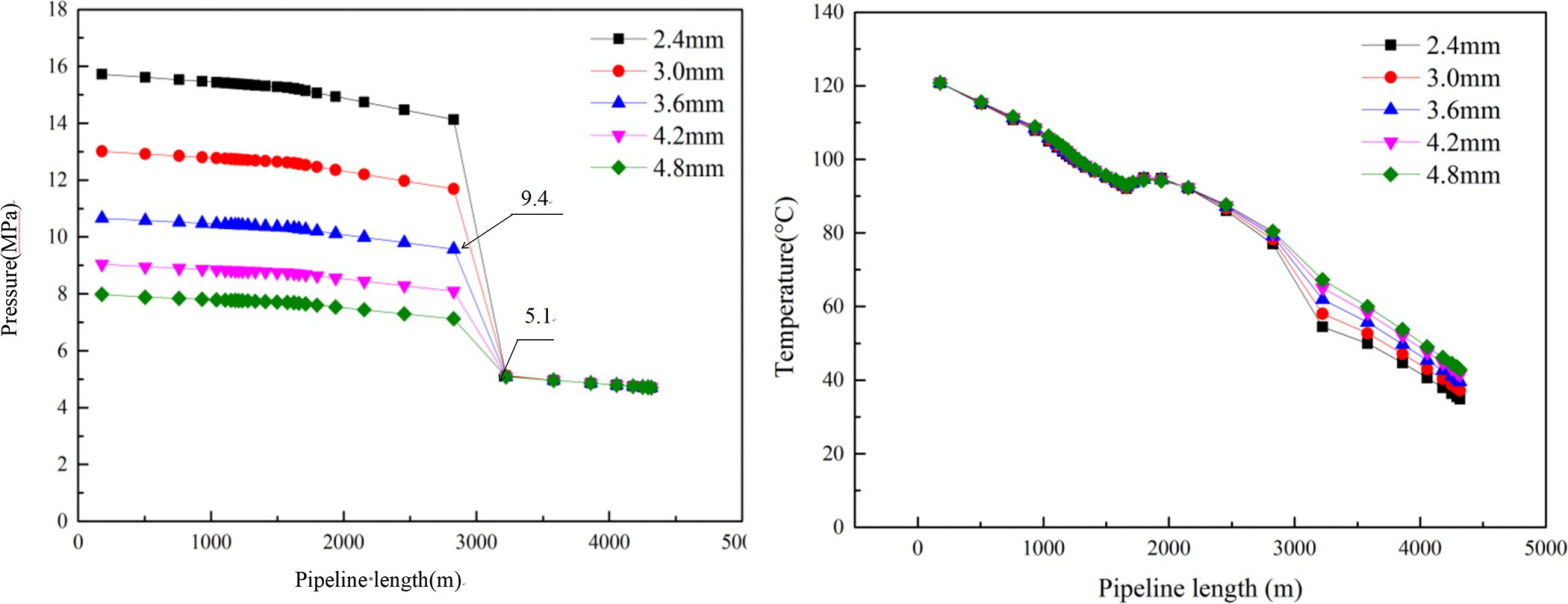
Pressure and temperature curves of choke with different nozzle diameters.

The calculation results are shown in Fig 5., in which, for example, in example 3 (where the throttle nozzle diameter is 3.6 mm), the pressure behind the throttle is 5.1MPa, the pressure before the throttle is 9.4MPa, the ratio is 0.542 less than 0.55, and the wellhead pressure can be reduced to 4.7MPa. The smaller the throttle diameter, the smaller the ratio between the pressure behind and before the throttle nozzle. However, the lower the temperature behind the throttle and the lower the temperature at the wellhead, the more readily hydrate is generated under the same pressure condition. Thus, combined with the critical flow theory, the diameter of the throttle was determined to be 3.6 mm.

**Fig 6:**
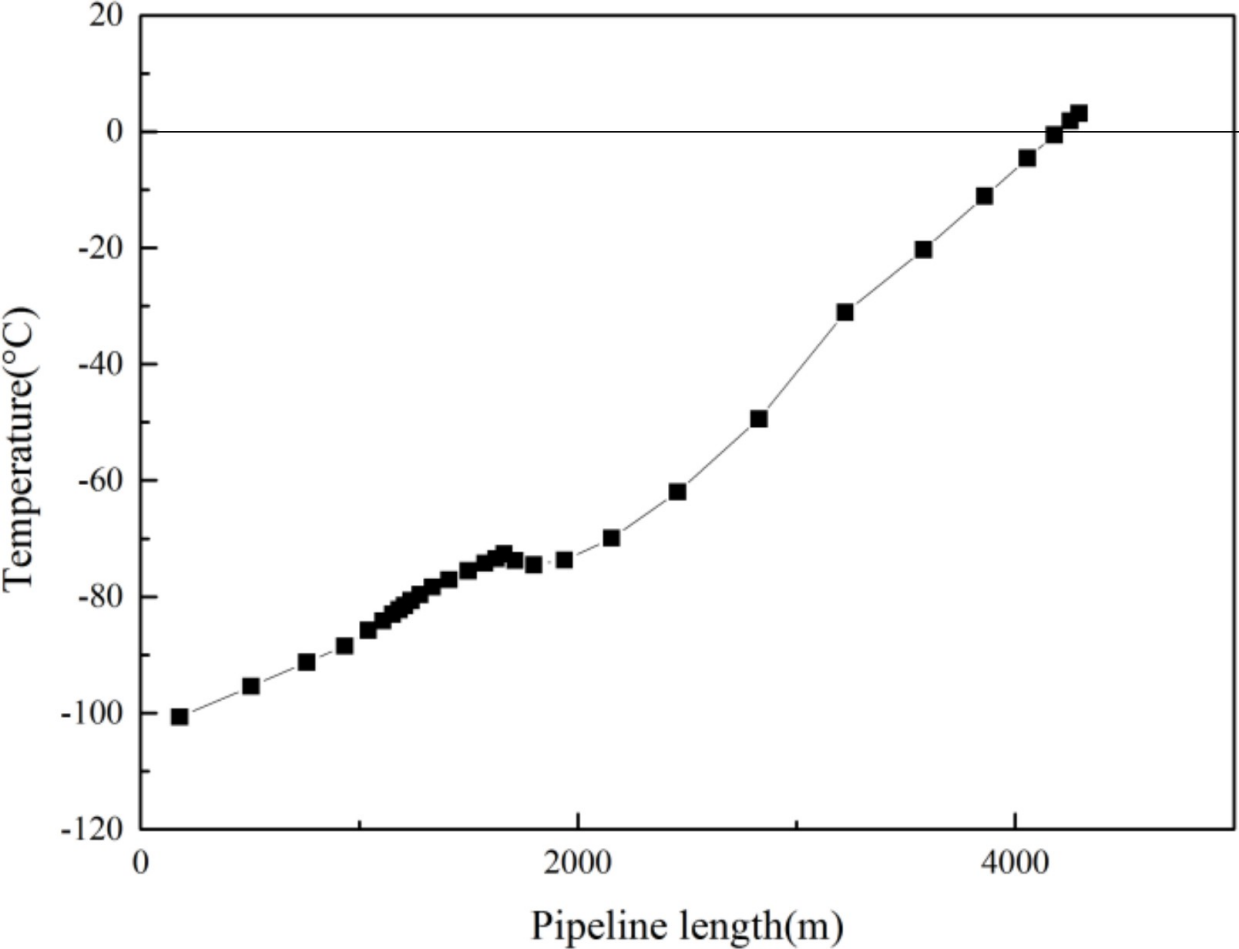
Temperature difference between hydrate and inner tubing section.

**Fig 7:**
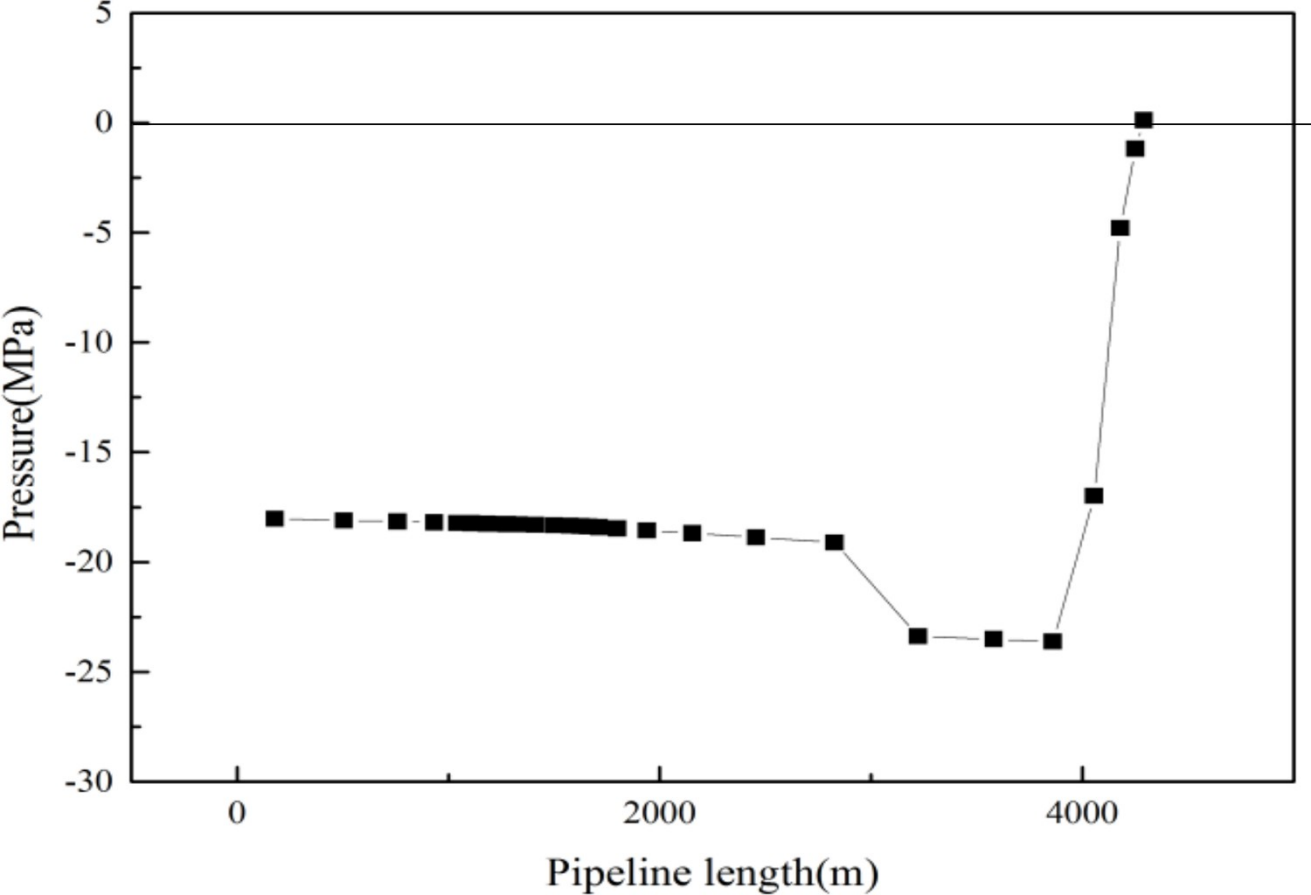
Pressure difference between hydrate and inner tubing section.

As can be seen in Figs 6 and 7, the temperature difference between the hydrate and the inner tubing section, and the pressure difference between the inner tubing section and the hydrate, respectively, were found to be greater than 0 within the range from wellhead to 148 m, indicating that hydrate will be generated within that depth range. Thus, when a downhole throttle with a nozzle diameter of 3.6 mm is lowered to the depth of the inner tubing of the gas well (1300 m), the wellhead oil pressure can be reduced, however, the formation of hydrate cannot be inhibited and, therefore, the addition of hydrate inhibitors would be necessary to reduce the temperature and, thus, inhibit the formation of hydrate.

**Fig 8:**
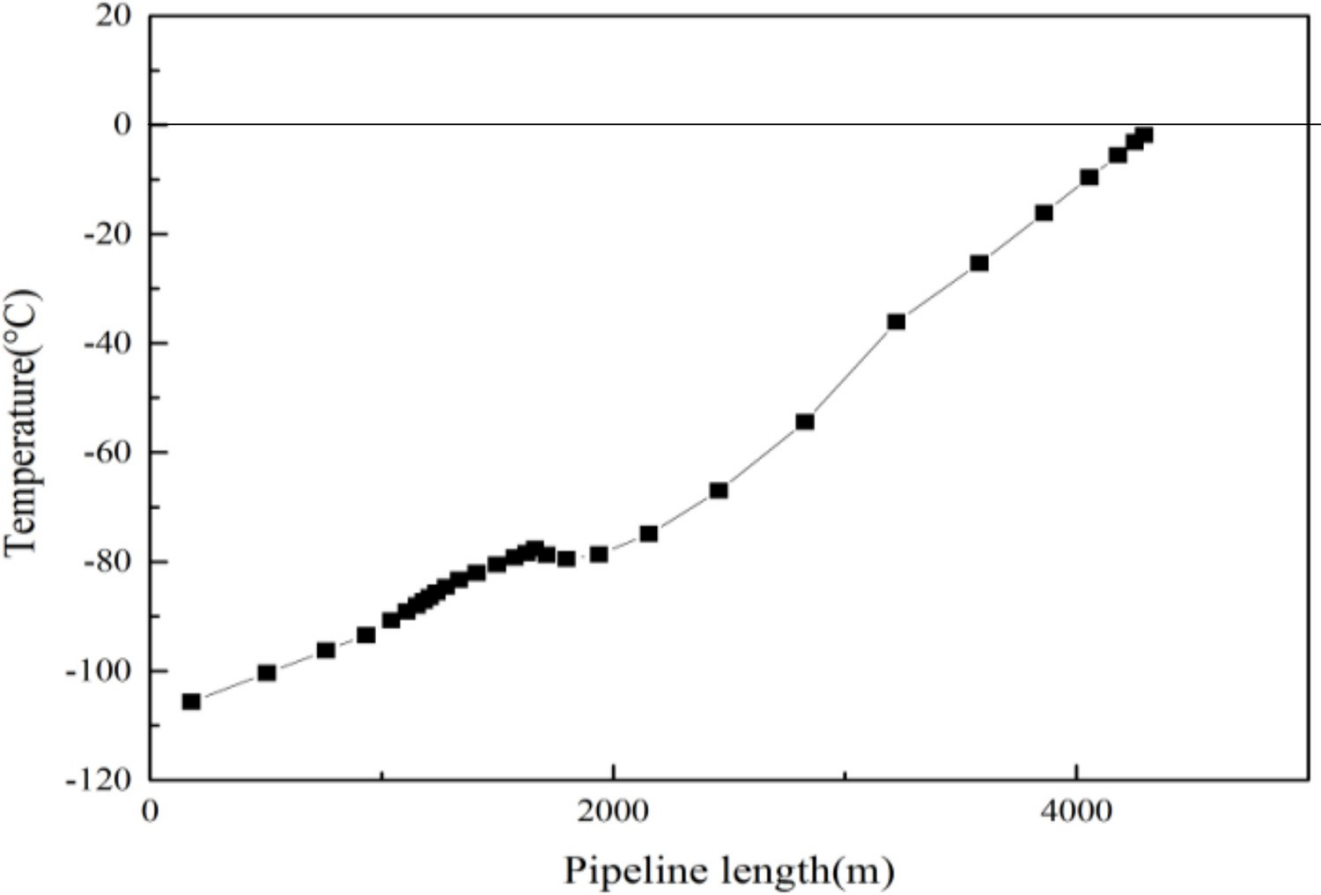
Temperature difference between hydrate and inner tubing section.

**Fig 9:**
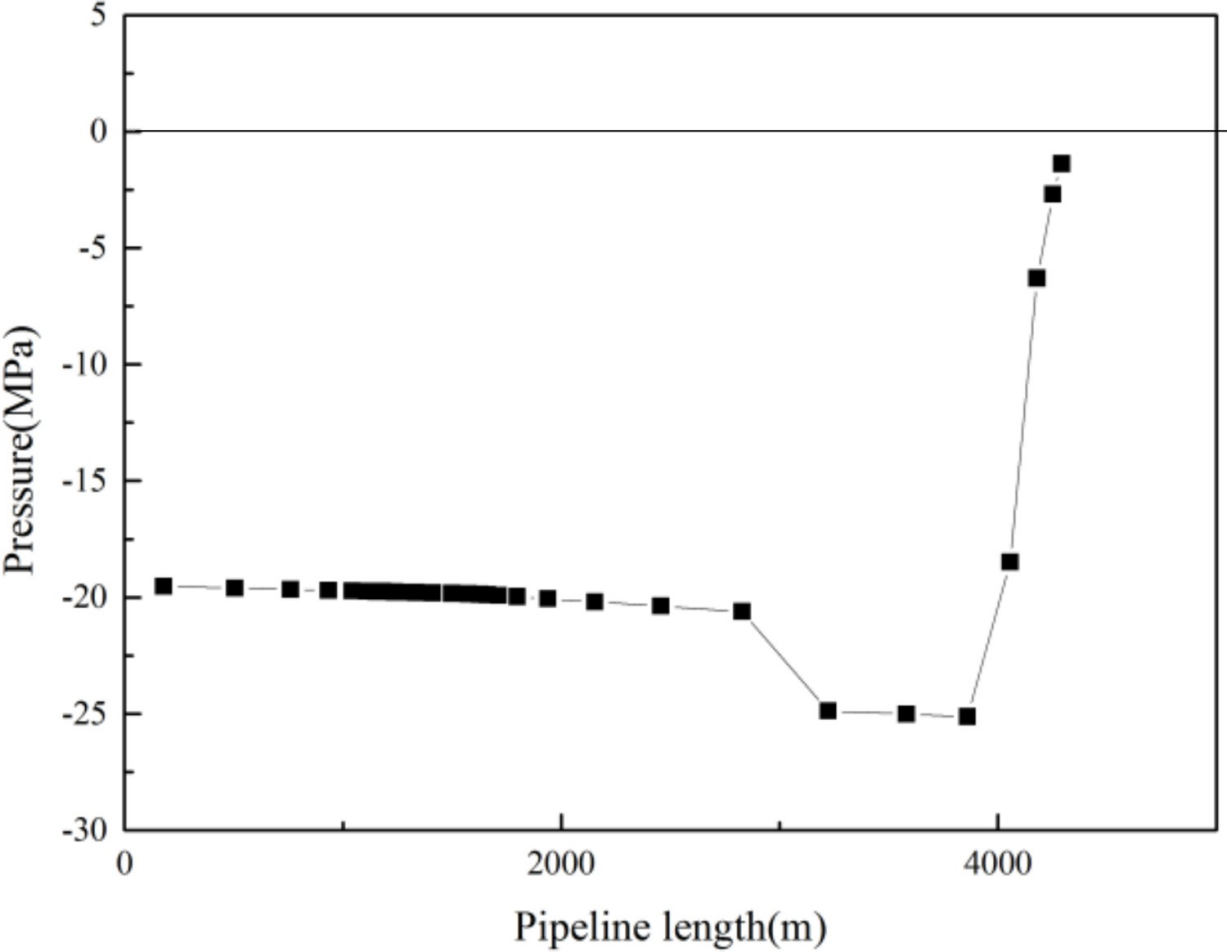
Pressure difference between hydrate and inner tubing section.

In general, the methanol content of hydrate inhibitors is greater than 30%, and methanol consumption is huge. Moreover, the use of hydrate inhibitors is affected by factors such as weather, personnel and equipment, and they cannot be continuously injected, which hinders the start-up rate of gas wells. Therefore, based on the downhole throttling process design results, the combination hydrate prevention and control process in this study was applied to optimize the filling concentration of hydrate inhibitors. According to the upper limit of 30% methanol content commonly used in gas Wells, hydrate inhibitors with a concentration of 30%, 25%, 20%, 15% and 5% methanol were used for simulation calculation, and the calculation results showed that the hydrate inhibitor with a concentration of 5% methanol could be used to effectively solve the problem of hydrate generation. As can be seen from Figs 8 and 9, after the addition of hydrate inhibitors with a 5% methanol concentration under the same conditions of depth and throttle nozzle diameter, the temperature difference between the hydrate and the inner tubing section, and the pressure difference between hydrate and the inner tubing section, were both less than 0 in the whole wellbore. Thus, when the downhole throttle with a 3.6 mm throttle nozzle was lowered to the depth of the gas well tubing at 1300 m, followed by the addition of the 5% methanol hydrate inhibitor, the wellhead oil pressure was reduced and the hydrate generation problem was solved.

### 3.3 Application effect of hydrate combination control process

**Fig 10:**
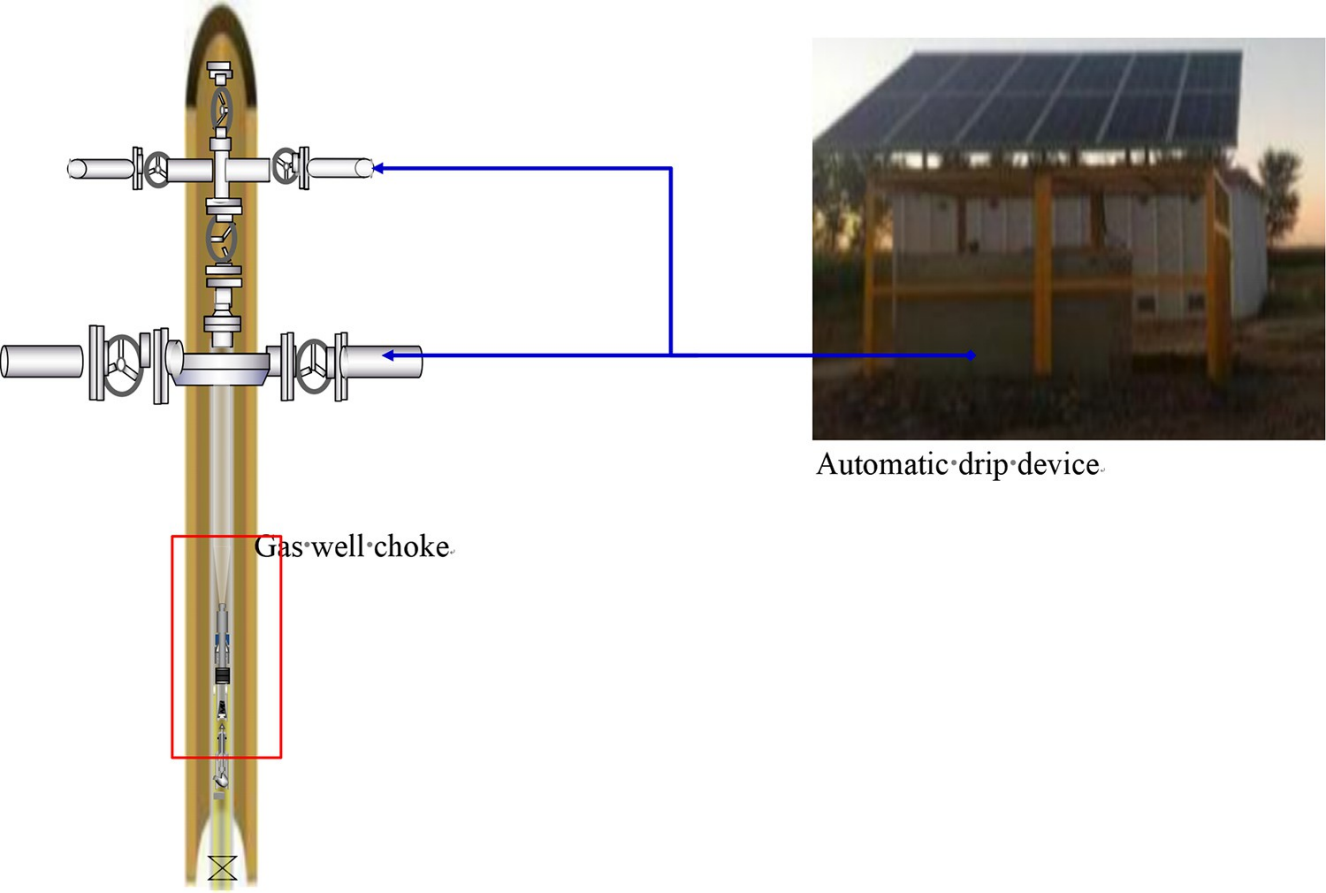
Schematic diagram of combination application of downhole choke and automatic drip device.

Using the design results of the combination hydrate control process parameters, this process was subsequently applied to Well H in the exploration of a new combined application, downhole throttling and surface hydrate inhibitor automatic drip device. The downhole throttling device was lowered into the gas well while the hydrate inhibitor automatic drip device was simultaneously installed at the wellhead, as shown in Fig 10.

**Fig 11:**
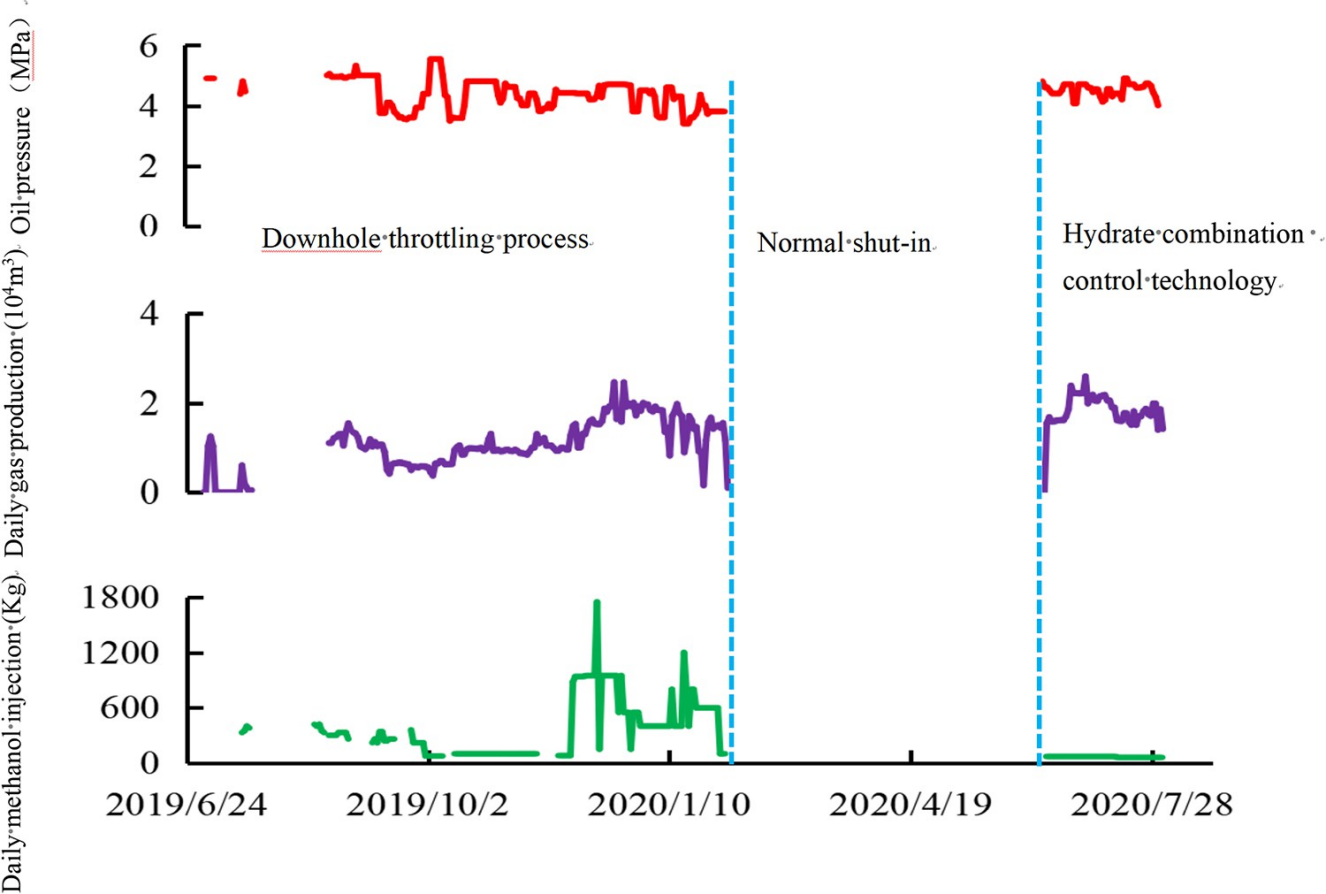
Production curve before and after well H test.

Fig 11 shows the production curve of Well H after the application of the combination hydrate control process. It was found that the downhole throttling process alone could reduce the wellhead oil pressure to an average of 5 MPa, however, a large amount of methanol (400–800 kg) had to be manually injected in order to maintain normal production. The combination approach of hydrate control technology, with downhole choke and the surface hydrate inhibitor automatic drip device, reduced the wellhead oil pressure to an average of 5 MPa and the methanol injection required from a maximum of 1750 kg to just 60 kg, thereby simultaneously achieving the reduction of oil pressure and inhibiting hydrate generation in the well. In addition, the automatic drip device of the hydrate inhibitor could automatically and continuously inject hydrate inhibitor without the need for manual maintenance under the normal production state of the gas well. In addition, the hydrate inhibitor automatic drip device can automatically and continuously inject hydrate inhibitors without manual maintenance under normal gas well production conditions, reducing manual maintenance workload by 80%, and increasing the rate of well opening from 45% to 100%.

## 4 Conclusions

1. Hydrate generation is affected mainly by wellbore temperature and pressure. The combined hydrate prevention and control technology proposed in this paper effectively solves the problem of wellbore throttling and depressurizing that leads to hydrate freezing and plugging in some wells. The management of temperature, pressure and well structure limitations, thus, provides technical means that contribute to the long-term stable and, even, increased production of such gas wells, as well as the reduction of well construction costs.
2. By using the novel combination hydrate control technology, the methanol injection volume was reduced in this study’s simulation from the maximum 1750 kg to just 60 kg, and the hydrate inhibitor could be automatically and continuously injected without manual maintenance, thereby significantly reducing methanol consumption and daily maintenance costs.

## Supporting information

S1 Data (XLSX)
